# Cardiovascular disease and thinning of retinal nerve fiber layer in a multi-ethnic Asian population: the Singapore epidemiology of eye diseases study

**DOI:** 10.3389/fmed.2023.1235309

**Published:** 2023-10-19

**Authors:** Shivani Majithia, Debra Q. Y. Quek, Miao Li Chee, Zhi Wei Lim, Simon Nusinovici, Zhi-Da Soh, Sahil Thakur, Tyler Hyungtaek Rim, Charumathi Sabanayagam, Ching-Yu Cheng, Yih-Chung Tham

**Affiliations:** ^1^Singapore Eye Research Institute, Singapore National Eye Centre, Singapore, Singapore; ^2^Yong Loo Lin School of Medicine, National University of Singapore, Singapore, Singapore; ^3^Ophthalmology and Visual Sciences Academic Clinical Program, Duke-NUS Medical School, Singapore, Singapore; ^4^Centre for Innovation and Precision Eye Health, Department of Ophthalmology, Yong Loo Lin School of Medicine, National University of Singapore, Singapore, Singapore

**Keywords:** retinal nerve fiber layer, ganglion cell-inner plexiform, cardiovascular disease, Asian, optical coherence tomography

## Abstract

**Introduction:**

Our study aimed to examine the relationship between cardiovascular diseases (CVD) with peripapillary retinal fiber layer (RNFL) and macular ganglion cell-inner plexiform layer (GCIPL) thickness profiles in a large multi-ethnic Asian population study.

**Methods:**

6,024 Asian subjects were analyzed in this study. All participants underwent standardized examinations, including spectral domain OCT imaging (Cirrus HD-OCT; Carl Zeiss Meditec). In total, 9,188 eyes were included for peripapillary RNFL analysis (2,417 Malays; 3,240 Indians; 3,531 Chinese), and 9,270 eyes (2,449 Malays, 3,271 Indians, 3,550 Chinese) for GCIPL analysis. History of CVD was defined as a self-reported clinical history of stroke, myocardial infarction, or angina. Multivariable linear regression models with generalized estimating equations were performed, adjusting for age, gender, ethnicity, diabetes, hypertension, hyperlipidaemia, chronic kidney disease, body mass index, current smoking status, and intraocular pressure.

**Results:**

We observed a significant association between CVD history and thinner average RNFL (β = −1.63; 95% CI, −2.70 to −0.56; *p* = 0.003). This association was consistent for superior (β = −1.79, 95% CI, −3.48 to −0.10; *p* = 0.038) and inferior RNFL quadrant (β = −2.14, 95% CI, −3.96 to −0.32; *p* = 0.021). Of the CVD types, myocardial infarction particularly showed significant association with average (β = −1.75, 95% CI, −3.08 to −0.42; *p* = 0.010), superior (β = −2.22, 95% CI, −4.36 to −0.09; *p* = 0.041) and inferior (β = −2.42, 95% CI, −4.64 to −0.20; *p* = 0.033) RNFL thinning. Among ethnic groups, the association between CVD and average RNFL was particularly prominent in Indian eyes (β = −1.92, 95% CI, −3.52 to −0.33; *p* = 0.018). CVD was not significantly associated with average GCIPL thickness, albeit a consistent negative direction of association was observed (β = −0.22, 95% CI, −1.15 to 0.71; *p* = 0.641).

**Discussion:**

In this large multi-ethnic Asian population study, we observed significant association between CVD history and RNFL thinning. This finding further validates the impact of impaired systemic circulation on RNFL thickness.

## Introduction

Glaucoma and cardiovascular diseases are among the top global concerns, given the increasing prevalence of these conditions and the associated burdens they impose (1, 2). Glaucoma is one of the leading causes of irreversible blindness (2), and CVD is one of the leading causes of mortality (1). Glaucoma is characterized by optic disc excavation and retinal nerve fiber layer (RNFL) thinning (3). The vascular theory of glaucoma proposes that impaired systemic circulation could compromise blood supply to the optic nerve, leading to optic nerve damage (4). In this regard, cardiovascular disease (CVD) has been hypothesized to be associated with open angle glaucoma (4–6).

Clinically, onset and progression of glaucoma is monitored by peripapillary RNFL thickness evaluation, and measured by spectral domain optical coherence tomography (SD-OCT) (7–11). In recent years, ganglion cell-inner plexiform layer (GCIPL) thickness measurement has also emerged as a vital clinical evaluation tool for glaucoma.

Notably, previous studies have reported equivocal findings pertaining to the relationship between CVD and RNFL thinning (12–15). The Gutenberg Health Study (3,224 eyes) demonstrated univariate associations between RNFL thickness with coronary artery disease and myocardial infarction (MI), but failed to replicate this association in a multivariable analysis (15). On the other hand, another study observed reduced RNFL thickness in patients with chronic heart failure (14). Furthermore, there is a lack of detailed research on the association between history of CVD and GCIPL thickness. Additionally, previous studies have primarily focused on examining the associations between cardiovascular risk factors such as elevated blood pressure, hypertension, and higher body mass index (BMI) with GCIPL thickness (16–18). Hence, the association between CVD with RNFL and GCIPL remains unclear.

To gain a deeper understanding of this aspect, a comprehensive evaluation on the association between CKD with RNFL and GCIPL thickness is warranted. Hence, utilizing the extensive multi-ethnic population-based Asian dataset from the Singapore Eye Epidemiology of Eye Diseases (SEED) study, we aimed to examine the relationships between CVD with RNFL and GCIPL thickness profiles.

## Materials and methods

### Study population and recruitment

Study participants were enrolled from the SEED study, comprising of the three major ethnic groups in Singapore (Malays, Indians, and Chinese). Details of the recruitment and methodology of the studies have been published previously (19). In brief, study participants aged 40 to 80 years were randomly sampled from the southwestern part of Singapore, using a standardized protocol across the three ethnic groups. Data for the current study were derived from the 6-year follow-up visits for the Malay (2011–2013, *n* = 1,901, response rate: 72.1%), Indian (2013–2015, *n* = 2,200, response rate: 75.5%), and Chinese cohorts (2015–2017, *n* = 2,661, response rate: 87.7%).

Written informed consent was obtained from all study participants. All study procedures were conducted in accordance to the tenets of the Declaration of Helsinki, and ethics approval was obtained from the SingHealth Centralized Institutional Review Board.

### Inclusion and exclusion criteria

Participants 40 years of age or older with complete SD-OCT data of RNFL and GCIPL thickness were included. Additionally, participants with relevant systemic, ocular, and lifestyle-related data such as the presence of hypertension, diabetes, hyperlipidemia, chronic kidney disease (CKD), CVD, smoking status, alcohol consumption and BMI were included. We excluded eyes with neurodegenerative diseases, and poor quality SD-OCT scans including poor signal strength (<6), segmentation errors, and ocular diseases affecting RNFL and GCIPL thickness.

### Ocular and systemic measurements

All participants underwent standardized systemic and ophthalmic examinations at the Singapore Eye Research Institute. Visual acuity and subjective refraction were measured by research optometrists. Intraocular pressure (IOP) was measured using a Goldmann applanation tonometer (Haag-Streit, Bern, Switzerland). Additionally, gonioscopy and a 24–2 SITA Fast Humphrey visual field (Humphrey Field Analyzer II; Humphrey Instruments, San Leandro, CA) test were performed for glaucoma suspects and participants with known glaucoma cases (diagnosed based on the International Society for Geographical and Epidemiological Ophthalmology, ISGEO guidelines) prior to dilation. Fundus examination was performed after pupil dilation with tropicamide 1% and phenylephrine 2.5%.

A detailed interviewer-administered questionnaire was used to collect relevant sociodemographic information and participant information including medication use, systemic and ocular history, current smoking status, and alcohol intake. CVD was defined as a self-reported history of stroke, MI, and/or angina. Non-fasting venous blood samples were collected for biochemical testing. Diabetes was defined as a random serum glucose ≥11.1 mmol/L or serum glycated hemoglobin ≥ 6.5%, use of diabetic medication and/or self-reported medical history of diabetes. Hypertension (HTN) was defined as a systolic blood pressure (BP) ≥ 140 mmHg, diastolic BP ≥ 90 mmHg, the use of anti-hypertensive medications and/or a self-reported medical history of hypertension. CKD was defined as an estimated glomerular filtration rate (eGFR) < 60 mL/min/1.73 m^2^. Hyperlipidemia was defined as total cholesterol ≥ 6.2 mmol/L, the use of lipid-lowering drugs and/or self-reported history of hyperlipidemia. Each participant’s weight was measured in kilograms using a digital scale, and height measured in centimeters using a wall-mounted measuring tape. BMI was calculated by the individual’s weight in kilograms divided by their height in meters squared.

### OCT imaging

**Figure 1:**
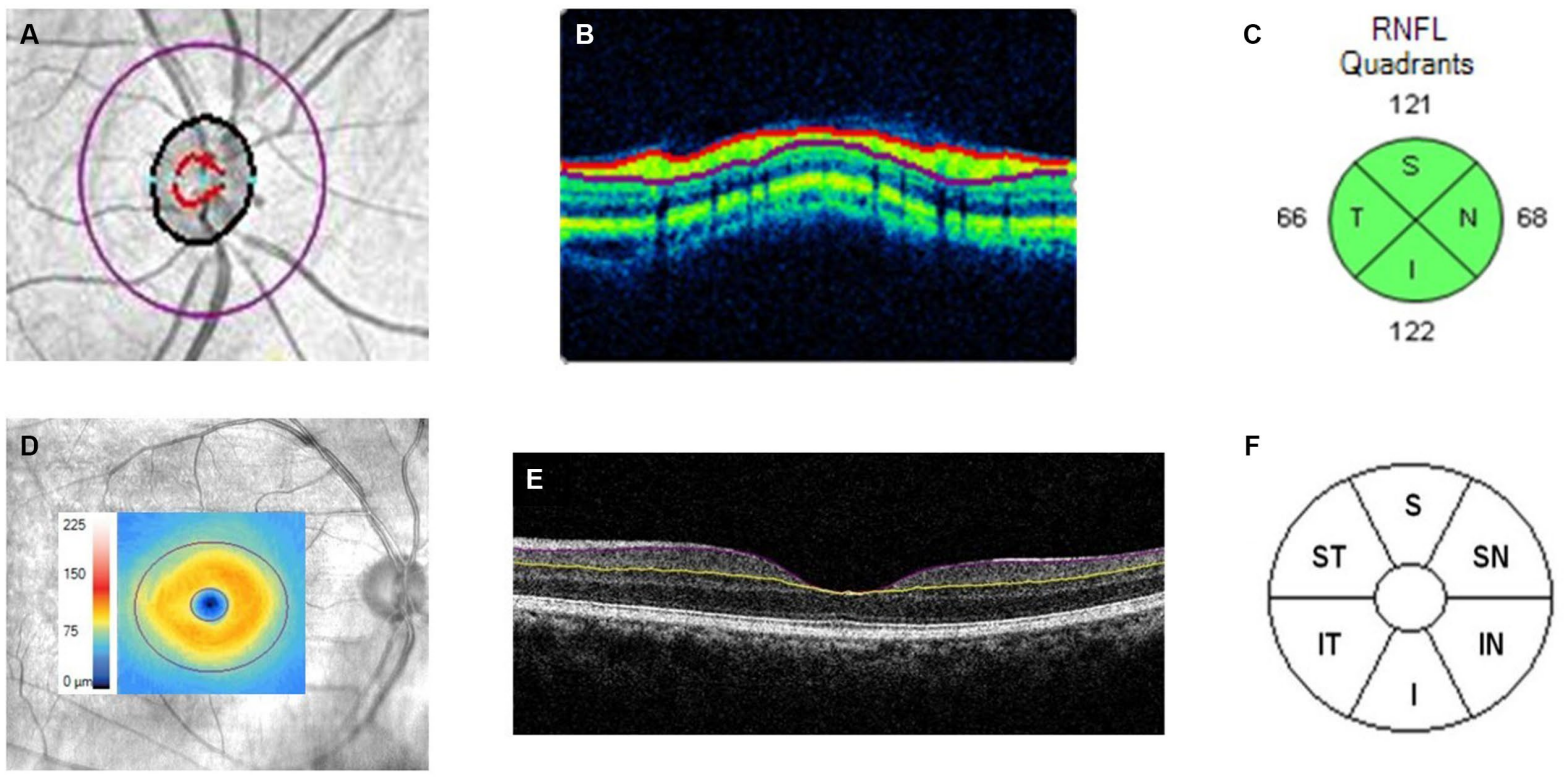
Cirrus HD-OCT images of the macular cube (512 × 128) and optic disc cube (200 × 200) scans of the right eye. **(A)** Fundus image of the optic nerve head with an overlay of the RNFL purple calculation circle. **(B)** Boundaries of RNFL segmentation (demarcated by the red and purple lines) extracted from the RNFL purple calculation circle. **(C)** Average thickness along RNFL purple calculation circle for superior (S), nasal (N), inferior (I), and temporal (T) quadrants. **(D)** Fundus image of the macula with an overlay showing a color-coded GCIPL thickness map within the 14.13 mm^2^ elliptical annulus area, centered on the fovea; **(E)** Single horizontal B scan of the macula showing segmentation of the GCIPL (boundaries of layer demarcated by the purple and yellow lines); **(F)** Division of the macular region into superior (S), superior-nasal (SN), superior-temporal (ST), inferior (I), inferior-nasal (IN), and inferior-temporal (IT).

For RNFL and GCIPL thickness measurements, OCT imaging was done after pupil dilation using Cirrus HD-OCT (Figure 1, Carl Zeiss Meditec, Dublin, CA). Optic disc scan was acquired using the optic disc cube 200 × 200 scan protocol, with a measurement area of 6 × 6 mm^2^. The optic nerve head and RNFL algorithms native to the Cirrus HD-OCT were used to measure the average and quadrant-specific peripapillary RNFL thickness automatically. For GCIPL thickness measurement, GCIPL measurements were acquired using the macular cube 512 × 128 scan protocol. An automated ganglion cell analysis algorithm incorporated into the Cirrus HD-OCT software version 6.5 was used to measure the average and sectoral GCIPL thickness. Further details about the measurement algorithms have been previously reported (20). In brief, for RNFL measurements, the algorithm automatically identifies Bruch’s membrane as the disc area, and the reference plane was determined 200 μm above the level of Bruch’s membrane plane. Additionally, for GCIPL measurements, the algorithm measured GCIPL thickness within a 14.13 mm^2^ elliptical annulus area that is centered on the fovea, and the posterior boundary of the RNFL and posterior boundary of the internal limiting membrane were automatically delineated by the ganglion cell analysis algorithm (21).

### Statistical analysis

All statistical analyses were performed using STATA statistical software (Version 15: StataCorp LP, College Station, TX). For descriptive statistics, the mean and standard deviation were reported for continuous variables, while frequency and percentages were reported for categorical variables. Associations between CVD with peripapillary RNFL and macular GCIPL parameters were examined using multivariable linear regression models with generalized estimating equation model to account for correlation between paired eyes of each subject. The models were adjusted for age, gender ethnicity, diabetes, hypertension, hyperlipidaemia, CKD, BMI, current smoking status, and IOP based on the rationale of potential confounders for RNFL and GCIPL thickness (13, 21, 22). *p*-value for significance was set at <0.05.

## Results

**Figure 2:**
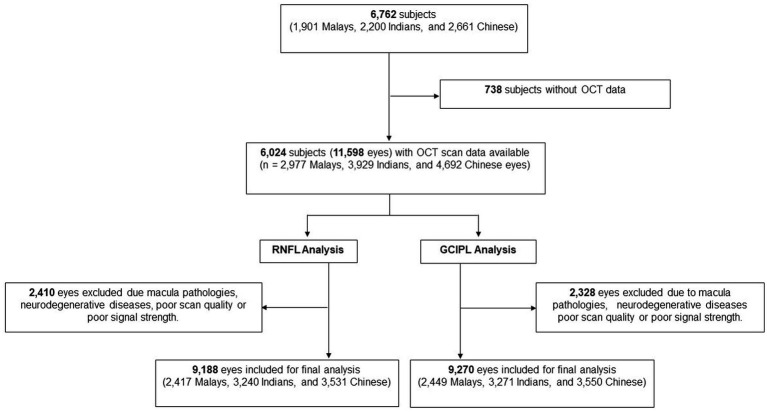
Flow chart of included participants.

Of the total 6,762 participants, 6,024 participants (11,598 eyes) had OCT scans done. After the exclusion of poor quality scans and retinal diseases which affect RNFL and GCIPL thickness, 9,188 eyes (2,417 Malays, 3,240 Indians, and 3,531 Chinese) were included for final peripapillary RNFL analysis and 9,270 eyes (2,449 Malays, 3,271 Indians, and 3,550 Chinese) for GCIPL analysis (Figure 2). The mean age of participants was 61.5 ± 8.5 years, and of them 51.8% were females (Table 1). The proportions of participants with systemic comorbidities were as follows: 28.2% of the participants had diabetes mellitus; 65.6% of the participants had hypertension; 8.8% of the participants had chronic kidney disease; and 8.8% of patients had CVD—of which, 2.0% had a stroke (27 Malays, 44 Indians, 34 Chinese), 5.3% had MI (80 Malays, 144 Indians, 54 Chinese), and 2.6% had angina (18 Malays, 90 Indians, 29 Chinese; Table 1).

**Table 1:** Characteristics of included participants.

Baseline characteristics	Mean (SD) or *n* (%)
Age, years	61.5 (±8.5)
Gender, female, *n* (%)	2,702 (51.8)
Current smoking, yes, *n* (%)	723 (13.9)
Alcohol consumption, yes *n* (%)	454 (8.8)
Diabetes mellitus, *n* (%)	1,470 (28.2)
HbA1c, %	6.1 (±1.2)
Random blood glucose, mmol/L	6.8 (±3.2)
Hypertension, *n* (%)	3,408 (65.6)
Systolic blood pressure, mmHg	137.5 (±19.5)
Diastolic blood pressure, mmHg	76.6 (±9.9)
Body mass index (BMI), kg/m^2^	25.5 (±4.6)
**BMI categories, *n* (%)**	
Underweight (BMI ≤ 18.5)	186 (3.6)
Normal (18.5 ≤ BMI ≤ 25)	2,426 (46.6)
Overweight (25 ≤ BMI ≤ 30)	1,836 (35.3)
Obese (30 ≥ BMI)	755 (14.5)
Total cholesterol, mmol/L	5.4 (±1.2)
High density lipoprotein cholesterol, mmol/L	1.3 (±0.3)
History of cardiovascular disease, *n* (%)	456 (8.8)
History of stroke, *n* (%)	105 (2.0)
History of myocardial infarction, *n* (%)	277 (5.3)
History of angina, *n* (%)	136 (2.6)
Chronic kidney disease, *n* (%)	435 (8.8)
Intraocular pressure, mmHg	14.8 (±2.9)
RNFL thickness, μm	91.3 (±11.4)
GCIPL thickness, μm	79.7 (±7.2)

RNFL, retinal nerve fiber layer; GCIPL, ganglion cell-inner plexiform layer. Data presented are mean (standard deviation) or frequency (proportion) variables.

After adjusting for age, gender, ethnicity, diabetes, hypertension, hyperlipidaemia, CKD, BMI, current smoking status, and IOP, we observed that a positive history of CVD was significantly associated with thinner average peripapillary RNFL (β = −1.63; 95% CI, −2.70 to −0.56; *p* = 0.003; Table 2). This association was consistent for superior (β = −1.79; 95% CI, −3.48 to −0.10; *p* = 0.038), inferior (β = −2.14; 95% CI, −3.96 to −0.32; *p* = 0.021), temporal (β = −1.39; 95% CI, −2.43 to −0.34; *p* = 0.013) and nasal (β = −1.12; 95% CI, −2.21 to −0.04; *p* = 0.043) quadrants (Table 2). Of the CVD types, a history of MI was observed to be particularly associated with thinner average RNFL (β = −1.75; 95% CI, −3.08 to −0.42; *p* = 0.010). This association was also consistently observed for superior (β = −2.22; 95% CI, −4.36 to −0.09; *p* = 0.041) and inferior RNFL quadrants (β = −2.42; 95% CI, −4.64 to −0.20; *p* = 0.033; Table 2).

**Table 2:** Association between cardiovascular disease and peripapillary retinal nerve fiber layer thickness.

		Peripapillary RNFL thickness (μm)^*^									
		Average		Superior quadrant		Inferior quadrant		Temporal quadrant		Nasal quadrant	
	Number of eyes	Beta (95% CI)	*p*-value	Beta (95% CI)	*p*-value	Beta (95% CI)	*p*-value	Beta (95% CI)	*p*-value	Beta (95% CI)	*p*-value
No CVD	8,421	Ref		Ref		Ref		Ref		Ref	
Presence of CVD	767	−1.63 (−2.70 to −0.56)	**0.003**	−1.79 (−3.48 to −0.10)	**0.038**	−2.14 (−3.96 to −0.32)	**0.021**	−1.39 (−2.43 to −0.34)	**0.010**	−1.12 (−2.21 to −0.04)	0.043
**Subtypes:**											
Stroke	171	−1.37 (−3.38 to 0.63)	0.180	−0.97 (−4.09 to 2.14)	0.539	−2.33 (−5.95 to 1.30)	0.208	−1.61 (−3.75 to 0.53)	0.140	−0.44 (−2.43 to 1.54)	0.661
Myocardial infarction	465	−1.75 (−3.08 to −0.42)	**0.010**	−2.22 (−4.36 to −0.09)	**0.041**	−2.42 (−4.64 to −0.20)	**0.033**	−1.12 (−2.42 to 0.17)	0.090	−1.09 (−2.47 to 0.29)	0.120
Angina	230	−0.93 (−2.85 to 1.00)	0.345	−0.85 (−3.80 to 2.11)	0.575	−0.25 (−3.38 to 2.88)	0.877	−1.35 (−3.17 to 0.46)	0.144	−1.17 (−3.09 to 0.75)	0.232

CVD, cardiovascular disease; RNFL, retinal nerve fiber layer. ^*^Model adjusted for age, gender, ethnicity, diabetes, hypertension, hyperlipidaemia, chronic kidney disease, body mass index, current smoking status, and intraocular pressure. Bold values indicate results that are statistically significant.

Table 3 shows the association between CVD and GCIPL thickness. After adjusting for age, gender, ethnicity, diabetes, hypertension, hyperlipidaemia, CKD, BMI, current smoking status, and IOP, no significant associations were observed, albeit a consistent negative direction of association was observed (β = −0.59; 95% CI, −1.33 to 0.15; *p* = 0.118). The non-significant association was also consistently observed for both superior and inferior hemispheres of GCIPL thickness (all *p* ≥ 0.066).

**Table 3:** Association between cardiovascular disease and macular ganglion cell inner plexiform layer thickness.

		Macular GCIPL thickness (μm)^*^					
		Average		Superior hemisphere		Inferior hemisphere	
	Number of eyes	Beta (95% CI)	*p*-value	Beta (95% CI)	*p*-value	Beta (95% CI)	*p*-value
No CVD	8,492	Ref		Ref		Ref	
Presence of CVD	778	−0.59 (−1.33 to 0.15)	0.118	−0.59 (−1.34 to 0.16)	0.124	−0.46 (−1.23 to 0.30)	0.237
**Subtypes:**							
Stroke	171	−1.34 (−2.83 to 0.14)	0.076	−1.42 (−2.94 to 0.10)	0.066	−1.19 (−2.73 to 0.35)	0.131
Myocardial infarction	475	−0.22 (−1.15 to 0.71)	0.641	−0.13 (−1.08 to 0.81)	0.786	−0.14 (−1.10 to 0.82)	0.774
Angina	233	−0.52 (−1.87 to 0.83)	0.450	−0.75 (−2.09 to 0.60)	0.278	−0.28 (−1.68 to 1.13)	0.700

CVD, cardiovascular disease; GCIPL, ganglion cell-inner plexiform layer. ^*^Model adjusted for age, gender, ethnicity, diabetes, hypertension, hyperlipidaemia, chronic kidney disease, body mass index, current smoking status, and intraocular pressure.

The association between CVD with average RNFL and GCIPL thickness by ethnicity are described in Table 4. After adjusting for age, gender, ethnicity, diabetes, hypertension, hyperlipidaemia, CKD, BMI, current smoking status, and IOP, CVD was associated with RNFL thinning in Indian eyes (β = −1.92; 95% CI, −3.52 to −0.33; *p* = 0.018). Additionally, among CVD subtypes, a history of MI was also associated with RNFL thinning in Indian eyes (β = −2.62; 95% CI, −4.55 to −0.69; *p* = 0.008). After adjusting for the same covariates, CVD showed a consistent lack of significant associated with GCIPL thinning across all ethnicities (all *p* ≥ 0.052).

**Table 4:** Association between cardiovascular disease with average peripapillary retinal nerve fiber layer and ganglion cell-inner plexiform layer thickness by ethnicity.

		Average RNFL (μm)^*^			Average GCIPL (μm)^*^	
	Number of eyes	Beta (95% CI)	*p*-value	Number of eyes	Beta (95% CI)	*p*-value
**Malay:**						
No CVD	2,223	Ref		2,249	Ref	
Presence of CVD	194	−1.31 (−3.09 to 0.48)	0.152	200	−0.26 (−1.42 to 0.90)	0.662
**Subtypes**						
Stroke	39	−1.37 (−4.55 to 1.81)	0.399	39	−1.13 (−3.48 to 1.22)	0.345
MI	137	−0.48 (−2.53 to 1.58)	0.649	143	0.01 (−1.33 to 1.36)	0.983
Angina	29	−4.25 (−9.27 to 0.77)	0.097	29	−0.04 (−3.14 to 3.06)	0.979
**Indian:**						
No CVD	2,838	Ref		2,865	Ref	
Presence of CVD	402	−1.92 (−3.52 to −0.33)	0.018	406	−0.88 (−1.99 to 0.23)	0.119
**Subtypes**						
Stroke	78	−1.43 (−4.80 to 1.95)	0.408	78	−2.46 (−4.94 to 0.03)	0.052
MI	246	−2.62 (−4.55 to −0.69)	0.008	249	−0.47 (−1.88 to 0.93)	0.510
Angina	154	−0.24 (−2.67 to 2.19)	0.846	157	−0.32 (−1.91 to 1.28)	0.696
**Chinese:**						
No CVD	3,360	Red		3,378	Red	
Presence of CVD	171	−1.62 (−3.89 to 0.65)	0.163	172	−0.26 (−1.93 to 1.41)	0.758
**Subtypes**						
Stroke	54	−1.29 (−4.95 to 2.38)	0.492	54	0.10 (−2.57 to 2.77)	0.942
MI	82	−1.69 (−5.14 to 1.76)	0.337	83	0.30 (−1.94 to 2.54)	0.793
Angina	47	−1.35 (−5.10 to 2.41)	0.482	47	−1.60 (−5.15 to 1.94)	0.376

CVD, cardiovascular disease; GCIPL, ganglion cell-inner plexiform layer; MI, myocardial infarction; RNFL, retinal nerve fiber layer. Model adjusted for age, gender, diabetes, hypertension, hyperlipidaemia, chronic kidney disease, body mass index, current smoking status, and intraocular pressure.

We further performed subgroup analyses on the association between CVD with RNFL and GCIPL thickness, excluding eyes with a diagnosis of glaucoma (Supplementary Tables 1, 2, respectively). After adjusting for age, gender, ethnicity, diabetes, hypertension, hyperlipidaemia, CKD, BMI, current smoking status, and IOP, we consistently observed that a positive history of CVD was significantly associated with thinner average (β = −1.44; 95% CI, −2.49 to −0.39; *p* = 0.007) and inferior quadrant peripapillary RNFL (β = −1.79; 95% CI, −3.56 to −0.01; *p* = 0.049). Additionally, among the different CVD subtypes, a history of MI was also significantly associated with average RNFL thinning (β = −1.67; 95% CI, −2.99 to −0.35; *p* = 0.013). Furthermore, after adjusting for the same covariates, CVD consistently demonstrated no significant association with GCIPL thinning in normal eyes (Supplementary Table 2).

## Discussion

In this large study of 6,762 multi-ethnic Asian participants, we observed a significant association between a history of CVD and RNFL thinning. This association remained significant after excluding eyes with a glaucoma diagnosis. The association of CVD with GCIPL was not significant albeit a consistent negative direction of association was observed. To the best of our knowledge, our study is the largest multi-ethnic Asian study in evaluating the associations between CVD with RNFL and GCIPL thickness profiles. Our findings further support the notion that systemic circulation is associated with RNFL thinning, indicating that a history of CVD may be taken into account when evaluating the risk of glaucoma.

Previous studies, which evaluated the association between CVD and RNFL thinning have reported non-conclusive findings (12, 13, 15, 23). While the Gutenberg Health Study reported no significant association between RNFL thickness and a history of coronary artery disease, MI, or stroke (15), the Asian Eye Epidemiology Consortium and European Eye Epidemiology Consortium observed that RNFL was 0.94 and 2.06 μm thinner in subjects with a history of CVD and stroke, respectively (13, 23). The latter two studies were consistent with our findings, which demonstrated an average decrease in RNFL thickness of 1.62 μm among participants with a history of CVD. This may be explained by microvascular ischemia commonly observed in participants with CVD (4), thereby further highlighting the relevance of CVD on RNFL thinning, which should be considered by clinicians when detecting and monitoring glaucoma.

Across ethnic groups in our study, we observed that the association between CVD and RNFL thinning was particularly prominent in Indian eyes, with an average RNFL decrease of 1.92 μm. This observation may, in part, be due to the fact that Indians have thinner RNFL profiles compared to Malay and Chinese populations (13, 22). The inherently thinner RNFL profile in Indian eyes could potentially make them more vulnerable to the adverse effects of reduced systemic circulation caused by CVD.

Following stratification for the types of CVD (stroke, MI, angina), we observed a significant association between MI and RNFL thinning. This may be partly explained by the systemic impacts of MI. MI results in ischaemic damage to the cardiac tissue, compromising cardiac ejection function and hence resulting in poorer perfusion of the optic nerve head and peripapillary region. On the other hand, the lack of association between stroke and angina with RNFL thinning in our study may potentially be due to the comparatively smaller sample size in the subgroups of stroke and angina (171 eyes with stroke and 230 eyes with angina, compared to 465 eyes with MI).

Coronary diseases and stroke are macrovascular processes associated with a limitation of blood supply to the heart or brain while retinal and retinal vasculature changes are microvascular processes (24). In macrovascular disease, the disease process is mainly due to atherosclerosis and atheroma formation (25). On the other hand, the disease process in microvascular disease involves multiple abnormalities in structure and function of the small vessels including reduced nitric oxide availability, reduced arteriolar diameter, and increased wall-to-lumen ratio of small arteries (24). Taken together, it is plausible that there are structural differences in the vasculature structures in the brain and heart, vs. the retina. However, the macrovasculature and microvasculature are an interconnected continuum, and changes in the macrovasculature circulation impacts the microvasculature circulation due to changes in blood delivery pace and flow. Nevertheless, among the CVD subtypes, we only observed a significant association between MI and RNFL thinning. To further elucidate the pathophysiological links between CVD subtypes and RNFL thinning, future studies utilizing OCT-angiography parameters or other CVD related biomarkers are warranted.

Contrary to our findings for RNFL thickness, a history of CVD showed no significant association with GCIPL thinning. We postulate that this may be due to the differential main blood supply of both layers. The RNFL layer receives its main vascular supply from the radial peripapillary capillary plexus, while the GCIPL is mainly perfused by the superficial vascular plexus (26). Given the difference in vascular supply, RNFL and GCIPL may also be differentially impacted by systemic circulatory changes, thus partially explaining the difference in association observed in this study.

The strengths of our study include a large sample size consisting of the three main ethnic groups (Malays, Indians, and Chinese) in Asia. Our comprehensive study protocol enabled us to consider an extensive list of potential confounding factors in our analysis. However, our study also has a few limitations. First, CVD status was based on participant’s self-reported history of MI, angina, or stroke. Nevertheless, as previous occurrence of a MI, angina, or stroke are likely to have been significant clinical events, self-reported error was less likely in this regard. Second, as our study is a cross-sectional study, we are unable to infer causality on the observed associations. A further longitudinal study would help to validate on the causal association between CVD and RNFL thinning.

In conclusion, we observed significant association between CVD and RNFL thinning in a large multi-ethnic Asian population. This association remained consistently significant even after excluding eyes with a diagnosis of glaucoma.

## Data availability statement

The raw data supporting the conclusions of this article will be made available by the authors, provided data access policy and ethics requirements are fulfilled.

## Ethics statement

The studies involving humans were approved by SingHealth Centralized Institutional Review Board. The studies were conducted in accordance with the local legislation and institutional requirements. The participants provided their written informed consent to participate in this study.

## Author contributions

SM, DQ, C-YC, and Y-CT conceived and designed the study. SM, DQ, MC, ZL, SN, Z-DS, ST, TR, and CS analyzed and interpreted the data. DQ, SM, MC, ZL, C-YC, and Y-CT wrote the manuscript. All authors contributed to the article and approved the submitted version.
