# Supplementary material for: Cardiovascular disease and thinning of retinal nerve fiber layer in a multi-ethnic Asian population: the Singapore epidemiology of eye diseases study
Source: Front Med (Lausanne). 2023 Oct 19;10:1235309. doi: 10.3389/fmed.2023.1235309 (PMC10620687; doi:10.3389/fmed.2023.1235309)
Supplement: Supplementary file 1 [file Table_1.DOCX]

**Supplementary Table 1: Association between cardiovascular disease and peripapillary retinal nerve fibre layer thickness in normal eyes**

|  |  | **Peripapillary RNFL Thickness (µm)*** | | | | | |
| --- | --- | --- | --- | --- | --- | --- | --- |
|  |  | **Average** | | **Superior Quadrant** | | **Inferior Quadrant** | |
|  | **Number of eyes** | **Beta (95% CI)** | **P value** | **Beta (95% CI)** | **P value** | **Beta (95% CI)** | **P value** |
| **No CVD** | 8,248 | Ref |  | Ref |  | Ref |  |
| **Presence of CVD** | 744 | -1.44 (-2.49 to -0.39) | **0.007** | -1.35 (-3.01 to 0.31) | 0.111 | -1.79 (-3.56 to -0.01) | **0.049** |
| **Subtypes:** |  |  |  |  |  |  |  |
| **Stroke** | 166 | -1.10 (-3.04 to 0.83) | 0.264 | -0.27 (-3.18 to 2.63) | 0.854 | -1.87 (-5.41 to 1.67) | 0.3 |
| **Myocardial infarction** | 451 | -1.67 (-2.99 to -0.35) | **0.013** | -2.08 (-4.20 to 0.04) | 0.055 | -2.16 (-4.31 to 0.00) | 0.05 |
| **Angina** | 225 | -0.70 (-2.56 to 1.16) | 0.461 | -0.38 (-3.22 to 2.47) | 0.794 | 0.03 (-3.01 to 3.06) | 0.986 |

Abbreviations: CVD: cardiovascular disease; RNFL: retinal nerve fiber layer

*Model adjusted for age, gender, ethnicity, diabetes, hypertension, hyperlipidaemia, chronic kidney disease, body mass index, current smoking status, and intraocular pressure.

**Supplementary Table 2: Association between cardiovascular disease and macular ganglion cell-inner plexiform layer thickness in normal eyes**

|  |  | **Macular GCIPL Thickness (µm)*** | | | | | |
| --- | --- | --- | --- | --- | --- | --- | --- |
|  | **Number of eyes** | **Average** | | **Superior Hemisphere** | | **Inferior Hemisphere** | |
|  |  | **Beta (95% CI)** | **P value** | **Beta (95% CI)** | **P value** | **Beta (95% CI)** | **P value** |
| **No CVD** | 8,317 | Ref |  | Ref |  | Ref |  |
| **Presence of CVD** | 754 | -0.45 (-1.18 to 0.28) | 0.226 | -0.46 (-1.20 to 0.28) | 0.227 | -0.32 (-1.07 to 0.43) | 0.400 |
| **Subtypes:** |  |  |  |  |  |  |  |
| **Stroke** | 166 | -1.25 (-2.73 to 0.24) | 0.101 | -1.27 (-2.77 to 0.23) | 0.098 | -1.14 (-2.70 to 0.42) | 0.150 |
| **Myocardial infarction** | 460 | -0.08 (-0.98 to 0.82) | 0.863 | -0.02 (-0.94 to 0.91) | 0.973 | 0.02 (-0.89 to 0.94) | 0.963 |
| **Angina** | 228 | -0.50 (-1.87 to 0.86) | 0.471 | -0.76 (-2.13 to 0.60) | 0.274 | -0.24 (-1.65 to 1.17) | 0.739 |

Abbreviations: CVD: cardiovascular disease; GCIPL: ganglion cell-inner plexiform layer; MI: myocardial infarction; RNFL: retinal nerve fiber layer

*Model adjusted for age, gender, ethnicity, diabetes, hypertension, hyperlipidaemia, chronic kidney disease, body mass index, current smoking status, and intraocular pressure.
